# *Momordica. charantia*-Derived Extracellular Vesicles-Like Nanovesicles Protect Cardiomyocytes Against Radiation Injury via Attenuating DNA Damage and Mitochondria Dysfunction

**DOI:** 10.3389/fcvm.2022.864188

**Published:** 2022-04-18

**Authors:** Wen-Wen Cui, Cong Ye, Kai-Xuan Wang, Xu Yang, Pei-Yan Zhu, Kan Hu, Ting Lan, Lin-Yan Huang, Wan Wang, Bing Gu, Chen Yan, Ping Ma, Su-Hua Qi, Lan Luo

**Affiliations:** ^1^Medical Technology School, Xuzhou Medical University, Xuzhou, China; ^2^Department of Laboratory Medicine, Affiliated Hospital of Xuzhou Medical University, Xuzhou, China; ^3^Department of Laboratory Medicine, Guangdong Provincial People's Hospital, Guangdong Academy of Medical Sciences, Guangzhou, China; ^4^Department of Rheumatology, The Second Affiliated Hospital of Nanchang University, Nanchang, China

**Keywords:** *Momordica. charantia*-derived extracellular vesicles-like nanovesicles, radiation-induced heart disease, DNA damage, mitochondria dysfunction, H9C2 cells

## Abstract

Thoracic radiotherapy patients have higher risks of developing radiation-induced heart disease (RIHD). Ionizing radiation generates excessive reactive oxygens species (ROS) causing oxidative stress, while *Momordica. charantia* and its extract have antioxidant activity. Plant-derived extracellular vesicles (EVs) is emerging as novel therapeutic agent. Therefore, we explored the protective effects of *Momordica. charantia*-derived EVs-like nanovesicles (MCELNs) against RIHD. Using density gradient centrifugation, we successfully isolated MCELNs with similar shape, size, and markers as EVs. Confocal imaging revealed that rat cardiomyocytes H9C2 cells internalized PKH67 labeled MCELNs time-dependently. *In vitro* assay identified that MCELNs promoted cell proliferation, suppressed cell apoptosis, and alleviated the DNA damage in irradiated (16 Gy, X-ray) H9C2 cells. Moreover, elevated mitochondria ROS in irradiated H9C2 cells were scavenged by MCELNs, protecting mitochondria function with re-balanced mitochondria membrane potential. Furthermore, the phosphorylation of ROS-related proteins was recovered with increased ratios of p-AKT/AKT and p-ERK/ERK in MCELNs treated irradiated H9C2 cells. Last, intraperitoneal administration of MCELNs mitigated myocardial injury and fibrosis in a thoracic radiation mice model. Our data demonstrated the potential protective effects of MCELNs against RIHD. The MCELNs shed light on preventive regime development for radiation-related toxicity.

## Introduction

With the advances in cancer management, clinicians have seen a remarkable improvement in cancer prognosis. The prolonged overall survival period has driven the recognition of cancer therapy-related adverse effects such as radiation-induced heart disease (RIHD) (1). As an essential modality for thoracic cancer therapy, radiotherapy (RT) eliminates the tumor, and injuries normal heart cells/tissues. The various heart cell types and structures have differential radiation sensitivity, leading to acute/chronic manifestations, including coronary artery atherosclerosis, valvular disease, pericarditis, conduction defects, and cardiomyopathy (2). Despite the recent development in RT strategy on the control of radiation doses and areas, the risks of RIHD are still unignorable (3). Of late, epidemiological studies have shown that atomic bomb survivors and Mayaka workers exhibited considerable RIHD risks years or decades after receiving low doses of radiation exposures (4, 5). Thus, the underlying mechanism that contributed to the occurrence of RIHD is urgent to comprehensive. Emerging experimental data has shown that radiation generates excessive reactive oxidative species (ROS), causing oxidative stress, epigenetic regulation, mitochondrial dysfunction, and telomere erosion in heart cells/tissues (6). These molecular effects further co-trigger endothelial dysfunction, inflammation, and fibrosis finally in the heart. However, the key determinants that result in the acute and chronic development of RIHD remain largely unidentified (6). Patients with acute RIHD have evident symptoms that are easy to diagnose and receive prompt medications. However, chronic RIHD is asymptomatic at the beginning and difficult to detect, which asks for harmless preventive regimes.

Various types of radioprotective agents, including synthetic chemicals, natural, and phytomedicine, have been discovered (7). Amifostine, the only Food and Drug Administration-approved chemical radioprotector has drawbacks of narrow administration routes and windows, high expenses, and inherent toxicities that minimize its efficacy (8). Recently, natural compounds have gained significant attention owing to their low expense, high accessibility, and less toxicity. It has proved that polyphenols, flavonoids, and various secondary metabolites extracted from different plant parts have radioprotective benefits via their anti-oxidation, DNA repair, anti-inflammation, signaling and apoptotic pathways modulation activities (7). Our group has been focusing on exploring the therapeutic effects of *Momordica. charantia* (*M. charantia*), a fruit of cucurbitaceae plant. We found that *M. charantia* extracts polysaccharides protected against cerebral ischemia/reperfusion injury via suppressing oxidative stress-mediated c-Jun N-terminal Kinase 3 signaling pathway (9). In addition, *M. charantia* polysaccharide promoted neural stem cells proliferation and differentiation via the SIRT1/Beta-catenin axis (10, 11). Considering the ROS scavenging activity of *M. charantia* polysaccharides, we wonder about its radioprotective capacity against RIHD.

Plant-derived extracts have disadvantages of large molecular mass, low solubility, difficulty in crossing physical barriers, and unknown complex composition limit their further applications (12). However, extracellular vesicles (EVs)-like nanovesicles innately derived from the plants could solve all these concerns and are novel candidates for their potential therapeutic benefits evaluation (13). Plant-derived EVs share similarities in size and content (proteins, lipids, DNAs, mRNAs, and microRNAs) of animal derived-EVs, mediating intercellular communications with mammalian cells (14). Chen et al. identified that exosome-like nanoparticles from *Ginger Rhizomes* inhibited NLRP3 inflammasome activation that holds premise for disease settings such as Alzheimer's disease and type 2 diabetes (15). Bruno et al. optimized a method to obtain *Citrus Sinensis*-EVs and found they modulated inflammatory genes and tight junctions in a human model of intestinal epithelium (16).

In this study, we first tried to isolate the *Momordica. charantia*-derived EVs-like nanovesicles (MCELNs) using density gradient centrifugation. We then identified MCELNs according to EVs' characterization criteria. Last, we investigated their protective effects against RIHD *in vitro* and *in vivo*.

## Materials and Methods

### Isolation of MCELNs

Fresh *M. charantia* (Kino Mountain, Xishuangbanna, Yunnan Province, China.) were gently washed with deionized water three times and then milled with the juice extractor. The *M. charantia* juice underwent a series of centrifugations as below. (1) 1,000 × g for 10 min, 3,000 × g for 20 min, 10,000 × g for 40 min at 4°C, the supernatant was kept; (2) 150,000 × g for 90 min at 4°C (Optima XE-90, Beckman Coulter Life Sciences, Indianapolis, U.S.), the pellet was kept; (3) the pellet was suspended with gradient sucrose (8%, 30%, 45%, 60%) and then centrifuged at 150,000 × g for 90 min at 4°C; (4) the band layer around 30–45% was washed with PBS and then centrifuged at 150,000 × g for 90 min at 4°C; (5) the pellet was suspended with PBS and passed through with a 0.22 μm filter (#SLGV004SL, Millipore) for further experiments or stored at −80°C. The BCA assay kit (#23235, Thermo Scientific) was performed to obtain the protein concentration of MCELNs.

### Transmission Electron Microscopy

MCELNs suspension (20 μl) was dropped onto Forvar carbon-coated grids (Electron Microscopy Sciences, Hatfield, PA, U.S.) for 3–5 min. Then stain with 2% phosphotungstic acid for 1–2 min at room temperature (RT) and take photos by transmission electron microscopy (FEI, Oregon, U.S.).

### Nanoparticle Tracking Analysis (NTA)

We examined the size distribution of MCELNs using a nanoparticle tracking analyzer (ZetaView, Bavaria, Germany). As the manual described, the MCELNs were diluted with PBS and then added to the analytical cell. The data of size distribution was obtained.

### Cell Culture and Radiation Exposure

The rat cardiomyocyte cell line (H9C2) was purchased from the National Collection of Authenticated Cell Cultures (Shanghai, China). H9C2 cells were cultured in Dulbecco's modified Eagle's High glucose (DMEM-H, C11995500BT, Gibco) supplemented with 10% fetal bovine serum (FBS, #10091-148, Gibco), 100 U/mL penicillin and 0.1 mg/mL streptomycin (#15140122, Gibco). Cells were maintained in a humidified incubator (37°C, 5% CO_2_, Heracell^TM^ 150i, Thermo Scientific).

Before radiation exposure, the culture medium was changed with DMEM-H medium supplemented with 10% exosome-depleted FBS (#EXO-FBS-50A-1, System Biosciences) and MCELNs. The cells were then exposed to 16 Gy X-ray at a dose rate of 200 cGy/min using X-RAD 225XL Biological Irradiator (Rad Source Technologies, North Branford, CT) as previously reported (17).

### Uptake of MCELNs by H9C2 Cells

MCELNs were labeled with the PKH67 Green Fluorescent Cell Linker Kit (#PKH67GL-1KT, Sigma-Aldrich) according to the manufacturer's protocol with minor modifications. In brief, MCELNs (10 μg/mL) diluted in PBS were added to 1 mL Diluent C. In parallel, 4 μl PKH67 dye was added to 1 mL Diluent C and incubated with the MCELNs solution for 4 min. Then, 2 mL 0.5% BSA/PBS was added to bind excess dye. PKH67-labeled MCELNs were washed with PBS and centrifugated at 150,000 × g for 90 min at 4°C. Finally, the pellet was diluted in PBS and went through with a 0.22 μm filter.

H9C2 cells (6.5 × 10^4^ cells/well) were seeded on a confocal dish and incubated with PKH67 labeled MCELNs for 6, 12, and 24 h at 37°C. Then cells were fixed with 4% paraformaldehyde, and the nuclei were stained with DAPI (#2031179, Invitrogen). Then, the images were taken by a confocal microscopy (STELLARIS 5 Confocal Microscope, Leica Microsystems, Illinois, U.S.).

### Cell Viability Assay

Cell viability was assessed by the Cell Proliferation Kit I (MTT assay, #11465007001, Roche Life Science). The H9C2 cells (5 × 10^3^ cells/well) were seeded in a 96-well culture plate. Cells were exposed to 0 or 16 Gy X-ray the next day. MCELNs (0, 0.5, 5, 10, and 25 μg/mL) were added to the culture medium before radiation exposure. After 48 h of culture, 10 μl MTT labeling solution was added to each well and incubated at 37°C for 4 h. Then, 100 μl solubilization solution was added to stop the formazan formation. Cells were cultured overnight and the absorbance at 570 nm was measured by an automatic microplate reader (Infinite F50, Tecan, Switzerland). The absorbance value of cells exposed to 0 Gy X-ray was included as a normalization control (%).

### Immunofluorescence Staining

The H9C2 cells (6.5 × 10^4^ cells/well) were seeded on the confocal dish. Cells were exposed to 0 or 16 Gy X-ray the next day. MCELNs (0 or 10 μg/mL) were added to the culture medium before radiation exposure. After 48 h of culture, cells were fixed with 4% paraformaldehyde for 15 min at RT. The cells were washed with PBS three times and incubated with 0.5% Triton X-100 for 10 min. Cells were blocked with 5% BSA at RT for 60 min. Then, cells were incubated with rat Ki-67 (#14-5698-82, Invitrogen), rabbit γ-H2A.x (#ab2893, Abcam), and p-ATM (#NB100-306, NOVUS) primary antibody at 4°C overnight, respectively. After washing with PBS three times, cells were incubated with associated fluorescent secondary antibody (#ab150158, Abcam; #A-11001, #A-11008, Thermo scientific) for 60 min at RT. The cells were then washed with PBS three times and stained with DAPI for 10 min. Images were captured by confocal microscopy (STELLARIS 5 Confocal Microscope, Leica Microsystems, Illinois, U.S.). Four fields from each group were selected randomly to calculate the proportion of Ki-67 positive stained cells relative to total cells.

### Western Blot Analysis

After indicated treatments, H9C2 cells were homogenized with ice-cold RIPA buffer (#89900, Thermo Scientific) containing 1% phosphatase and protease inhibitors (#78442, Thermo Scientific). Total protein concentration was measured by BCA assay kit (#23225, Thermo Scientific). The proteins were separated on SDS-PAGE gels (7.5%, #1610181, Bio-Rad; 12%, #1610185, Bio-Rad) and transferred to 0.22 μm PVDF membranes (#1620177, Bio-Rad) via Trans-Blot^®^ Turbo^TM^ transfer system (Bio-Rad, California, U.S.). Membranes were blocked with 5% BSA in Tris Buffered Saline with Tween 20 (TBST) for 1 h at RT and then incubated with the primary antibody at 4°C overnight. The primary antibodies included TSG101 (#28283-1-AP, proteintech), CD63 (#25682-1-AP, proteintech), CD9 (#20597-1-AP, proteintech), PCNA (#A0264, ABclonal), Cyclin D1 (#2978, CST), Cyclin B1 (#55004-1-AP, proteintech), cleaved caspase3 (#9664, CST), cleaved PARP (#9548, CST), γ-H2A.x (#ab2893, Abcam), ATM (#NB100-309, NOVUS), p-ATM (#NB100-306, NOVUS), AKT (#60203-2-lg, proteintech), p-AKT (#4060, CST), ERK (#BF8004, affinity), p-ERK (#AF1015, affinity), β-actin (#66009-1-lg, proteintech), α-tubulin (#66031-1-lg, proteintech). After sufficient washing with TBST, membranes were incubated with HRP-conjugated secondary antibodies for 1 h at RT. Blots were visualized using an ECL detection kit (#RPN2135, GE Healthcare Life Sciences) using a ChemiDoc^TM^ Touch imaging system (Bio-Rad, California, U.S.). The density of the band was analyzed by the Image lab software (Bio-Rad, California, U.S.).

### Apoptosis Assay

H9C2 cells apoptosis was assessed using PE Annexin V apoptosis detection kit (#559763, BD Biosciences). Cells were exposed to 0 or 16 Gy X-ray the next day. MCELNs (0 or 10 μg/mL) were added to the culture medium before radiation exposure. After 48 h of culture, the cells were harvested with 0.25% trypsin without EDTA (#15090046, Gibco) and washed with PBS three times. Cells (1 × 10^5^ cells) were resuspended in 100 μl of Binding Buffer. Then, 5 μl of Annexin V-PE and 5 μl of 7-AAD were added to the cell suspensions at RT in the dark. After incubation for 15 min, the cells were added with 400 μl ice-cold binding buffer. Cell apoptosis rate was analyzed using a flow cytometer within 1 h (FACS Canto II, Becton Dickinson, New Jersey, U.S.). Q3 and Q2 area represnted the early and late cell apoptosis, respectively. The total cell apoptosis rate was the sum of Q3 and Q2 apoptosis rate.

### Measurement of Mitochondria ROS Generation

Mitochondria ROS were detected by MitoSOX kit (#M36008, Invitrogen). H9C2 cells were exposed to 0 or 16 Gy X-ray the next day. MCELNs (0 or 10 μg/mL) were added to the culture medium before radiation exposure. After 48 h of culture, cells were harvested with 0.25% trypsin without EDTA and washed with PBS three times. Then, cell pellets were resuspended with 1 mL of 5 μM MitoSOX™ reagent working solution and incubated for 10 min at 37°C in the dark. After washing with PBS three times, cells were analyzed using a flow cytometer (FACS Canto II, Becton Dickinson, New Jersey, U.S.).

### Measurement of Mitochondrial Membrane Potential (MMP, ΔΨm)

Mitochondrial Membrane Potential Detection Kit (JC-1, #abs50016-100T, absin) was used. H9C2 cells were exposed to 0 or 16 Gy X-ray the next day. MCELNs (0 or 10 μg/mL) were added to the culture medium before radiation exposure. After 48 h of culture, cells were harvested with 0.25% trypsin without EDTA and washed with PBS three times. Then, cells were added with 0.5 mL DMEM and 0.5 mL prepared JC-1 working solution. After incubation for 20 min at 37°C in the dark, cell suspensions were washed with JC-1 staining buffer two times. Then resuspend cell pellets with JC-1 staining buffer and analyzed using flow cytometer (FACS Canto II, Becton Dickinson, New Jersey, U.S.).

### Thoracic Mice Irradiation Model

5-6 weeks BALB/c nude mice (GemPharmatech Co., Ltd) weighed about 18–22 g were used in this experiment. This study was approved by the Institutional Animal Care and Use Committee of Xuzhou Medical University (202112A020), and all animal procedures were performed following the institutional and national guidelines.

Animals were randomly divided into three groups (*n* = 4): (1) Control group; (2) IR Group; (3) MCELNs administration post-IR (100 μg/kg every other day for 5 times, intraperitoneal injection). Thoracic radiation exposure was performed in mice at a dose rate of 100 cGy/min, 20 Gy X-ray using X-RAD 225XL Biological Irradiator (Rad Source Technologies, North Branford, CT) as previously reported (18). We covered the other body areas except the chest with lead to shield from the X-ray. The body weight was weighed every two days, and mice were sacrificed 35 days after the initial thoracic radiation exposure. Serum was collected for cardiac injury biomarkers evaluation. Heart weight was measured and then fixed with 4% paraformaldehyde for paraffin sections preparation (4 μm).

### Enzyme-Linked Immunosorbent Assay (ELISA)

The mouse serum were used to detect myocardial injury biomarkers with ELISA kits, including CK-MB (#E-EL-M0355c, Elabscience), cTnT (#E-EL-M1801c, Elabscience), and NT-proBNP (#E-EL-M0834c, Elabscience).

### Masson Trichrome Staining

The cardiac tissues were sectioned in 4 μm for Masson trichrome staining according to the mannual (#G1006-20ML, Servicebio). The images were observed and scanned using a microscope inspection (Olympus VS120). The fibrosis area were analyzed using Image-J software (1.52n).

### Statistical Analysis

All experiments are presented as the mean ± SD. The statistical significance was evaluated by one-way analysis of variance (ANOVA) followed by Turkey's multiple comparisons test among groups (GraphPad Prism 9.3.0). RM two-way ANOVA followed by Turkey's multiple comparisons test was used for significance evaluation of repeat measurement of mice body weight. Differences were considered significant when *P* < 0.05.

## Results

### Isolation and Characterization of MCELNs

**Figure 1:**
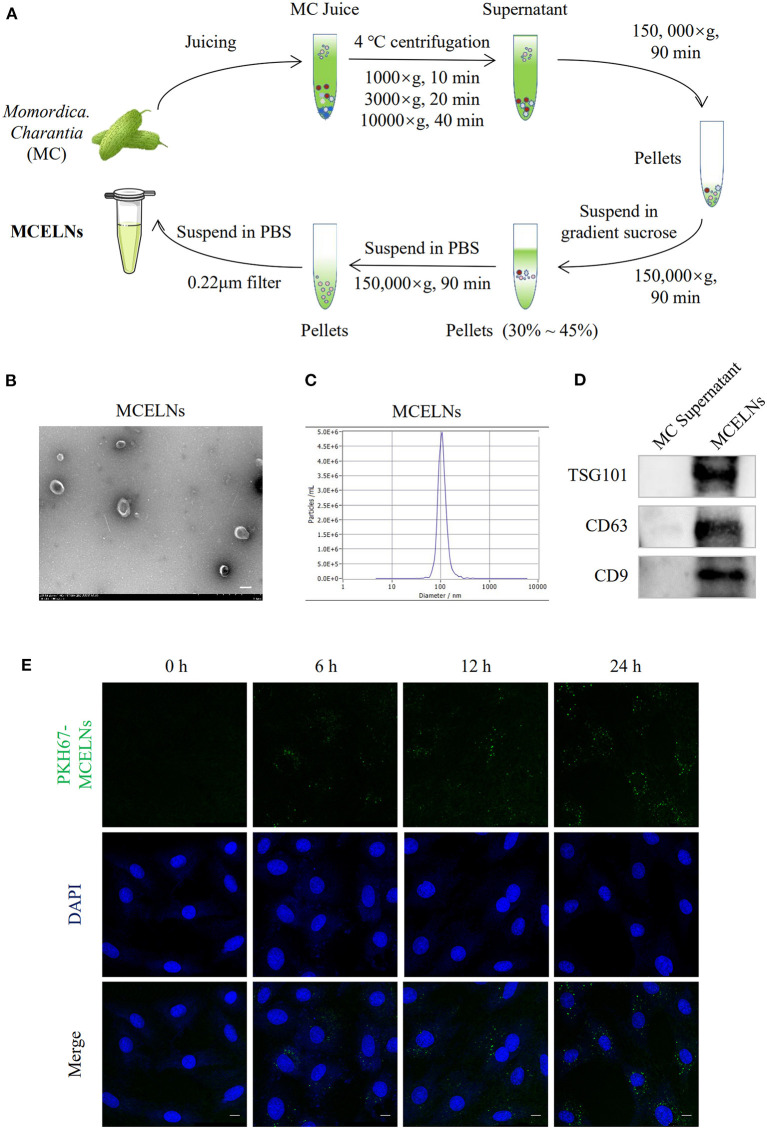
Isolation and characterization of MCELNs. **(A)** Flow sheet on the isolation of MCELNs from *Momordica. charantia* (MC) using gradient density centrifugation. **(B)** Transmission electron microscope image of MCELNs, scale bar: 2 μm. **(C)** NTA analysis exhibited the diameter of MCELNs was around 106.0 nm. **(D)** Western blot analysis of EVs biomarker, including TSG101, CD63, and CD9 in MC supernatant and MCELNs. **(E)** Representative images on the uptake of PKH67-labled MCELNs (green) in H9C2 cells when co-cultured at 0, 6, 12, 24 h. The nucleus were stained with DAPI (blue), scale bar: 10 μm.

Figure 1A illustrates the flow chart of MCELNs isolation from *M. charantia* (MC). Transmission electron microscopy imaging revealed that MCELNs exhibited a cup-shaped morphology (Figure 1B). Using NTA assay, MCELNs had an average diameter of 106.0 nm, similar to animal-derived EVs (Figure 1C). Western blot assay showed higher expressions of EVs marker (TSG101, CD63, and CD9) in MCELNs than the supernatant obtained during the isolation process (Figure 1D). These data demonstrated the successful isolation of MCELNs characterized as EVs. Next, we labeled MCELNs with fluorescent membrane dyes PKH67 and evaluated the uptake of MCELNs in H9C2 cells at 0, 6, 12, 24 h. Confocal imaging showed that H9C2 cells internalized PKH67-MCELNs in a time-dependent manner (Figure 1E).

### MCELNs Promote the Proliferation of H9C2 Cells After Radiation Exposure

**Figure 2:**
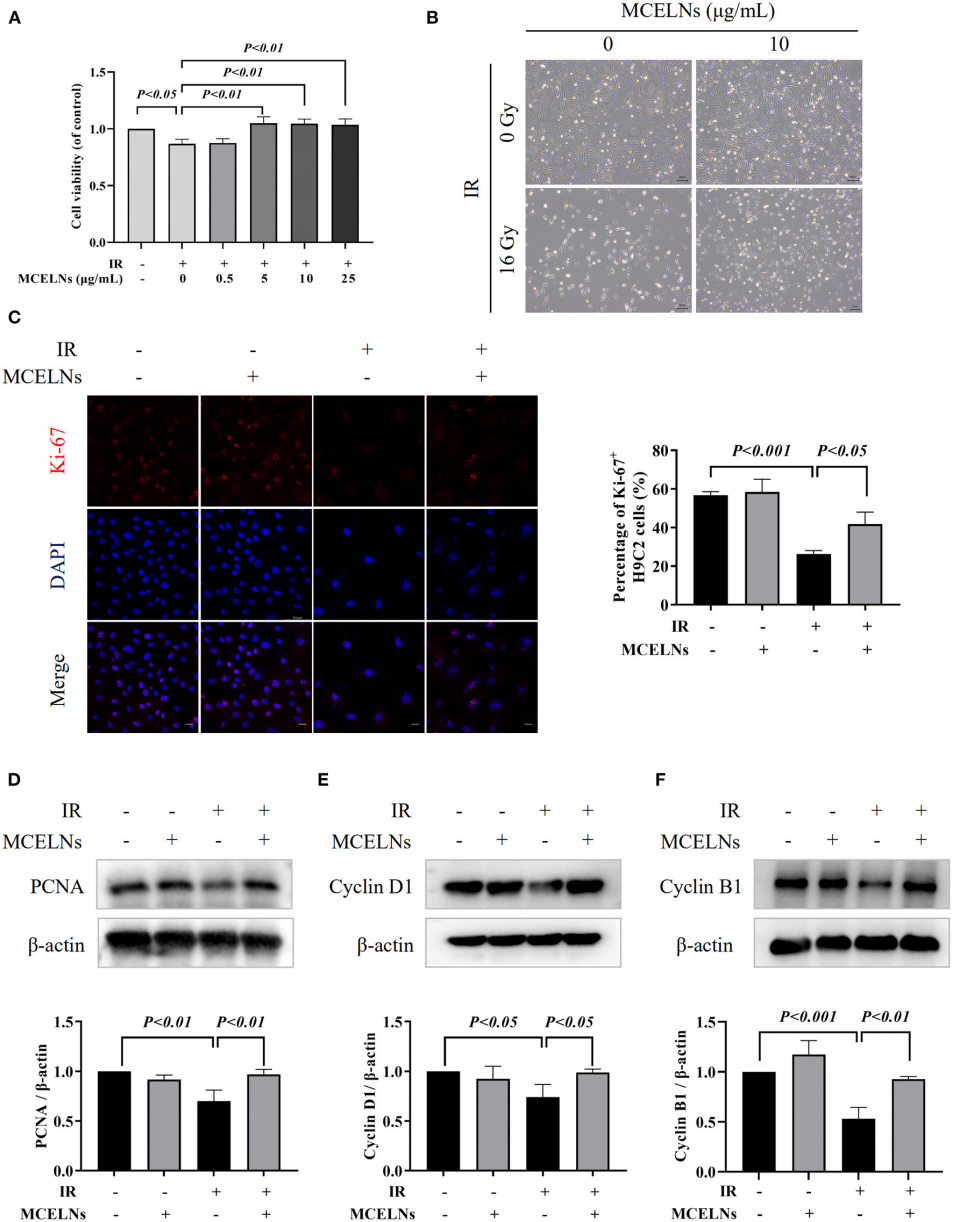
MCELNs enhanced the proliferation of H9C2 cells after radiation exposure. **(A)** H9C2 cells were previously treated with different doses of MCELNs (0, 0.5, 5, 10, 25 μg/mL) and then exposed to 16 Gy X-ray. After 48 h of culture, the cell viability of H9C2 cells was determined using a MTT assay. **(B)** Representative growth images of H9C2 cells after 48 h of culture with indicated treatment, scale bar: 200 μm. **(C)** Immunofluorescence staining (left) and quantitation (right) of Ki-67 (red) positive stained H9C2 cells after 48 h of culture with indicated treatment. The nucleus were stained with DAPI (blue), scale bar: 10 μm. Western blot analysis and quantitation on the expressions of PCNA **(D)**, Cyclin D1 **(E)**, and Cyclin B1 **(F)** in H9C2 cells after 48 h of culture with indicated treatment. IR (–/+): 0/16 Gy X-ray; MCELNs (–/+): 0/10 μg/mL. All data were represented as means ± SD (*n* = 3 independent experiments). The statistical significance was evaluated by one-way ANOVA followed by the Turkey's multiple comparisons test among groups.

Firstly, we evaluated whether MCELNs could improve cell proliferation activity in irradiated H9C2 cells. H9C2 cells were previously treated with different doses of MCELNs (0, 0.5, 5, 10, 25 μg/mL) and then exposed to 16 Gy X-ray. After 48 h of culture, the MTT assay identified that MCELNs mitigated radiation-induced decreased cell viability in H9C2 cells dose-dependently (Figure 2A). We included 10 μg/mL of MCELNs for further experiments. Cell growth of irradiated or non-irradiated H9C2 cells treated with MCELNs (10 μg/mL) or not after 48 h culture were shown in Figure 2B. By the immunofluorescence staining of cell proliferation marker Ki-67, we identified a significantly decreased percentage of Ki-67 positive H9C2 cells after exposure to 16 Gy X-ray (Figures 2C,D). However, MCELNs (10 μg/mL) dramatically increased the percentage of Ki-67 positive H9C2 cells (Figure 2C). We further investigated the expressions of proliferation marker PCNA (Figure 2D) and cell cycle-related proteins, including Cyclin D1 (Figure 2E) and Cyclin B1 (Figure 2F) in H9C2 cells after indicated radiation exposure and MCELNs treatment. Western blot assay showed that MCELNs (10 μg/mL) recovered the downregulated expressions of PCNA, Cyclin D1, and Cyclin B1 in irradiated H9C2 cells. These data revealed that the MCELNs promoted H9C2 cells proliferation after radiation exposure.

### MCELNs Suppress the Apoptosis of H9C2 Cells After Radiation Exposure

**Figure 3:**
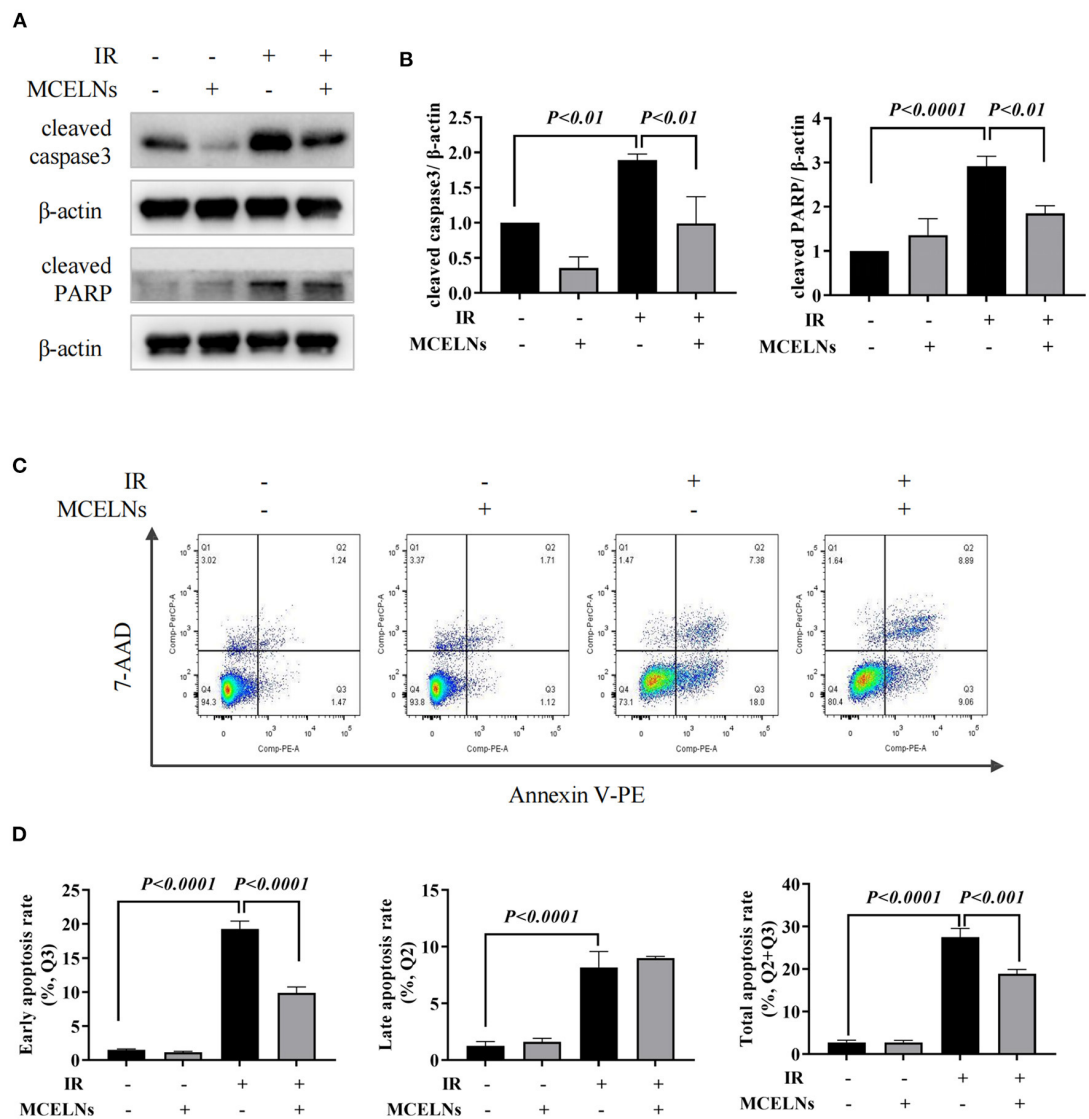
MCELNs inhibited the apoptosis of H9C2 cells after radiation exposure. Western blot images **(A)** and quantitation **(B)** of apoptotic proteins cleaved-caspase3 and cleaved-PARP in H9C2 cells after 48 h of culture with indicated treatment. Annexin V apoptosis Flow detection **(C)** and quantitation **(D)** of the apoptosis rate in H9C2 cells after 48 h of culture with indicated treatment. IR (–/+): 0/16 Gy X-ray; MCELNs (–/+): 0/10 μg/mL. All data were represented as means ± SD (*n* = 3 independent experiments). The statistical significance was evaluated by one-way ANOVA followed by the Turkey's multiple comparisons test among groups.

We next explored the protective effects of MCELNs on the apoptosis of H9C2 cells against radiation using western blot and Annexin-V flow cytometry assay. MCELNs (10 μg/mL) significantly reduced the expressions of apoptosis proteins cleaved caspase3 and cleaved PARP (Figures 3A,B) that were elevated in irradiated H9C2 cells. Flow cytometry assay showed that exposure to 16 Gy X-ray significantly enhanced the apoptosis of H9C2 cells, and MCELNs (10 μg/mL) significantly reduced the total apoptosis rate of H9C2 cells, especially the early apoptosis rate (Figures 3C,D). These data verified that MCELNs suppressed the apoptosis of H9C2 cells after radiation exposure.

### MCELNs Mitigate the DNA Damage of H9C2 Cells After Radiation Exposure

**Figure 4:**
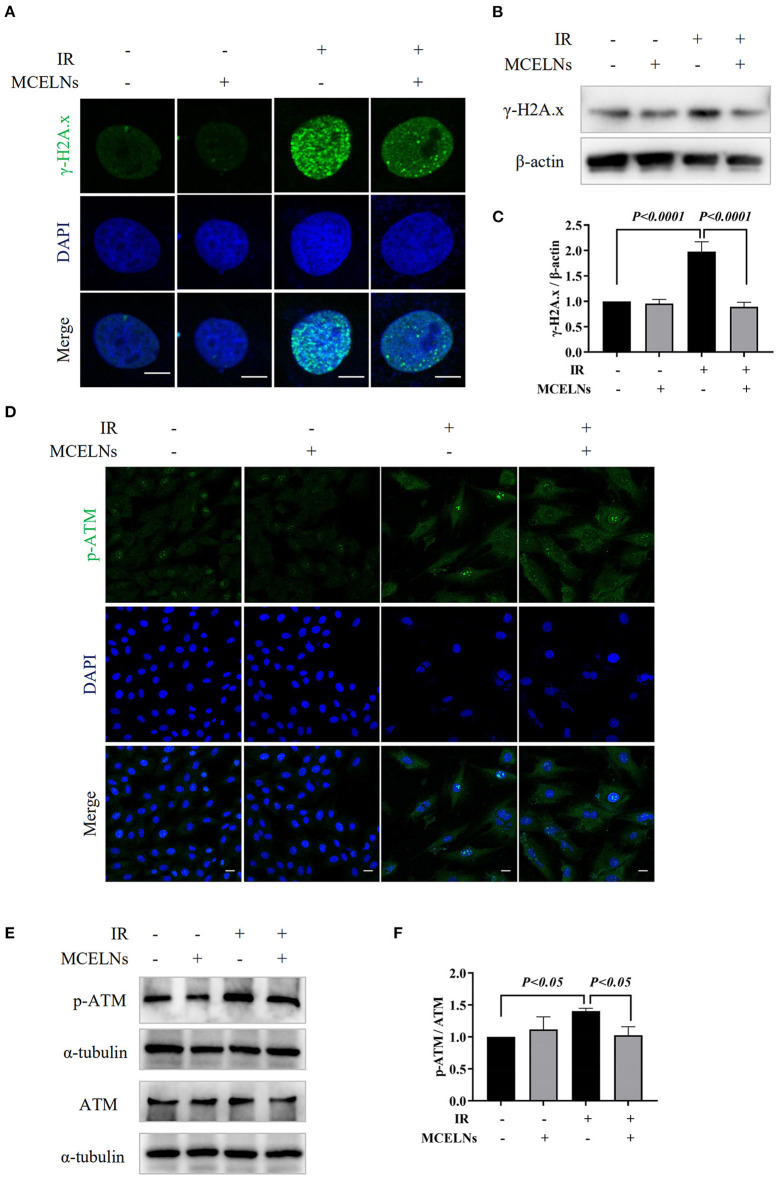
MCELNs decreased the DNA damage of H9C2 cells after radiation. **(A)** Immunofluorescence staining of γ-H2A.X (green) in H9C2 cells after 48 h of culture with indicated treatment. The nucleus were stained with DAPI (blue), scale bar: 10 μm. Western blot images **(B)** and quantitation **(C)** of γ-H2A.X in H9C2 cells after 48 h of culture with indicated treatment. **(D)** Immunofluorescence staining of p-ATM (green) in H9C2 cells after 48 h of culture with indicated treatment. The nucleus were stained with DAPI (blue), scale bar: 10 μm. Western blot images **(E)** and quantitation **(F)** of p-ATM and ATM in H9C2 cells after 48 h of culture with indicated treatment. IR (–/+): 0/16 Gy X-ray; MCELNs (–/+): 0/10 μg/mL. All data were represented as means ± SD (*n* = 3 independent experiments). The statistical significance was evaluated by one-way ANOVA followed by the Turkey's multiple comparisons test among groups.

The direct effect caused by radiation is DNA damage. Here, we tested whether MCELNs could alleviate the DNA damage of H9C2 cells after radiation exposure. Immunofluorescent staining revealed that exposure to 16 Gy X-ray caused a substantial accumulation of DNA damage marker γ-H2A.X foci in the nucleus of H9C2 cells (Figure 4A). Western blot assay further confirmed a significantly elevated protein expression of γ-H2A.X in irradiated H9C2 cells (Figures 4B,C). MCELNs (10 μg/mL) significantly reduced γ-H2A.X foci formation in the nucleus (Figure 4A) and its protein expression (Figures 4B,C). The protein kinase ataxia-telangiectasia mutated (ATM) is an apical activator involved in the phosphorylation of the DNA double-strand breaks damage response pathway. After DNA double-strand breaks, ATM was gathered to DNA damage sites and phosphorylated to regulate DNA damage response, especially γ-H2A.X phosphorylation. Immunofluorescent staining showed that MCELNs (10 μg/mL) declined the expressions of p-ATM in the nucleus of irradiated H9C2 cells (Figure 4D). Western blot assay further proved these changes (Figures 4E,F). Thus, MCELNs protected the H9C2 cells against DNA damage after radiation exposure.

### MCELNs Attenuate the Mitochondrial Dysfunction in H9C2 Cells After Radiation Exposure

**Figure 5:**
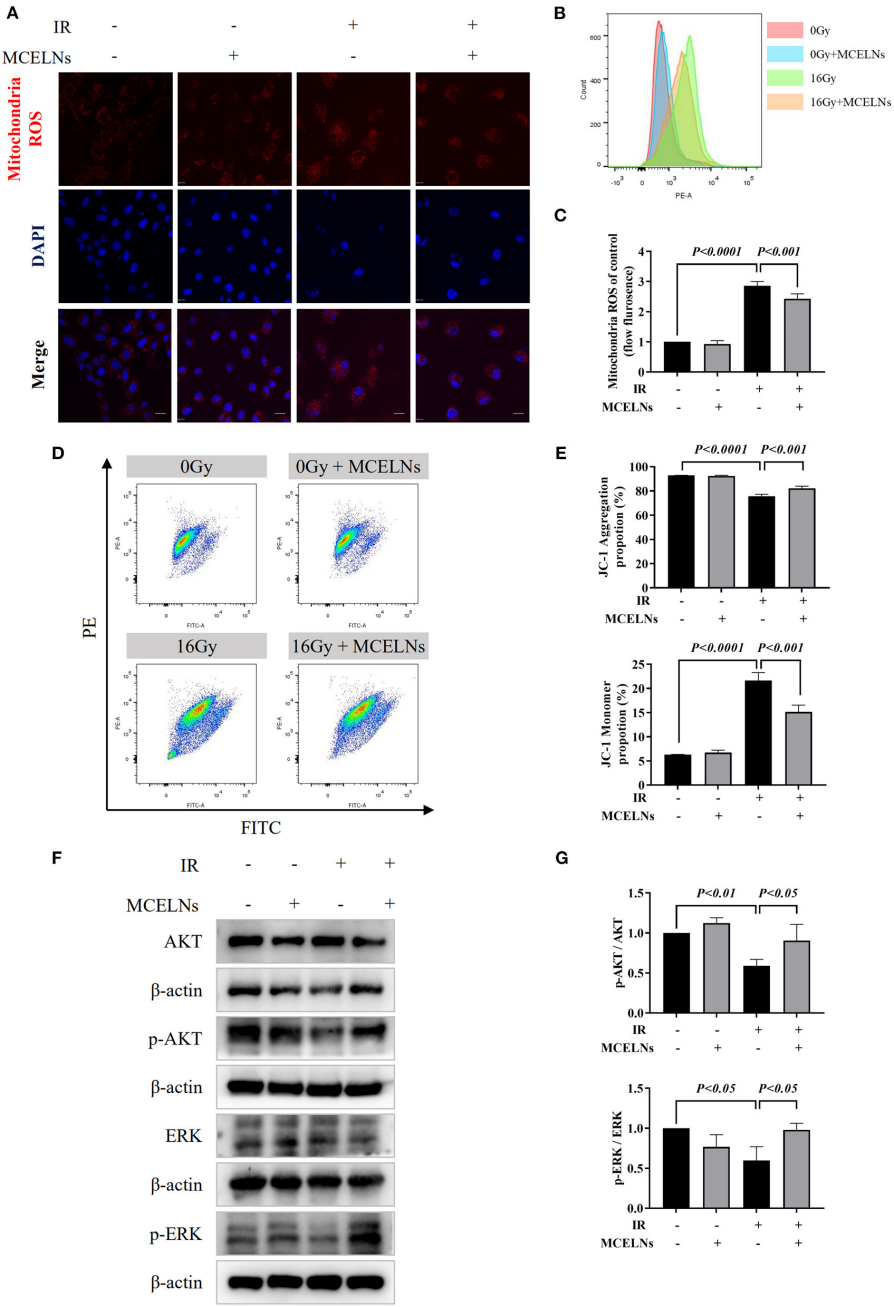
MCELNs ameliorated mitochondrial dysfunction induced cell injury after radiation. **(A)** H9C2 cells after 48 h of culture with indicated treatment were incubated with mitochondria ROS probe (red) for 15 min. Then nucleus were stained with DAPI (blue) for confocal imaging, scale bar: 20 μm. Flow analysis **(B)** and quantitation **(C)** of mitochondria ROS intensity in H9C2 cells after 48 h of culture with indicated treatment. Flow analysis **(D)** and quantitation **(E)** of mitochondria membrane potential in H9C2 cells after 48 h of culture with indicated treatment that detected by JC-1 probe. Western blot images **(F)** and quantitation **(G)** of AKT, p-AKT, ERK, and p-ERK in H9C2 cells after 48 h of culture with indicated treatment. IR (–/+): 0/16 Gy X-ray; MCELNs (–/+): 0/10 μg/mL. All data were represented as means ± SD (*n* = 3 independent experiments). The statistical significance was evaluated by one-way ANOVA followed by the Turkey's multiple comparisons test among groups.

Radiation exposure causes cell injury by excessive ROS generation, and mitochondria are the primary source of intracellular ROS. Hence, we tested the mitochondria ROS levels and quality after indicated radiation exposure and MCELNs treatment. Mitochondria ROS staining (Figure 5A) and flow cytometry assay (Figures 5B,C) demonstrated that exposure to 16 Gy X-ray triggered excessive mitochondria ROS generation in H9C2 cells. MCELNs (10 μg/mL) significantly diminished the mitochondria ROS levels in irradiated H9C2 cells (Figures 5A–C). To evaluate the mitochondria quality, we tested the MMP (ΔΨm) of H9C2 cells using the JC-1 probe. After radiation exposure, the proportion of JC-1 monomer significantly increased and JC-1 aggregation decreased, indicating reduced MMP (Figures 5D,E). MCELNs (10 μg/mL) suppressed radiation-induced JC-1 monomer generation and augmented JC-1 aggregation proportion, thus recovering MMP (Figures 5D,E). Last, we evaluated the ROS-related proteins, including p-AKT, AKT, p-ERK, and ERK. MCELNs significantly upregulated the reduction in the ratio of p-AKT/AKT and p-ERK/ERK in irradiated H9C2 cells (Figures 5F,G). These data demonstrated that MCELNs could attenuate the mitochondrial dysfunction in H9C2 cells after radiation exposure.

### MCELNs Exerted a Cardioprotective Effect in a Thoracic Mice Irradiation Model

**Figure 6:**
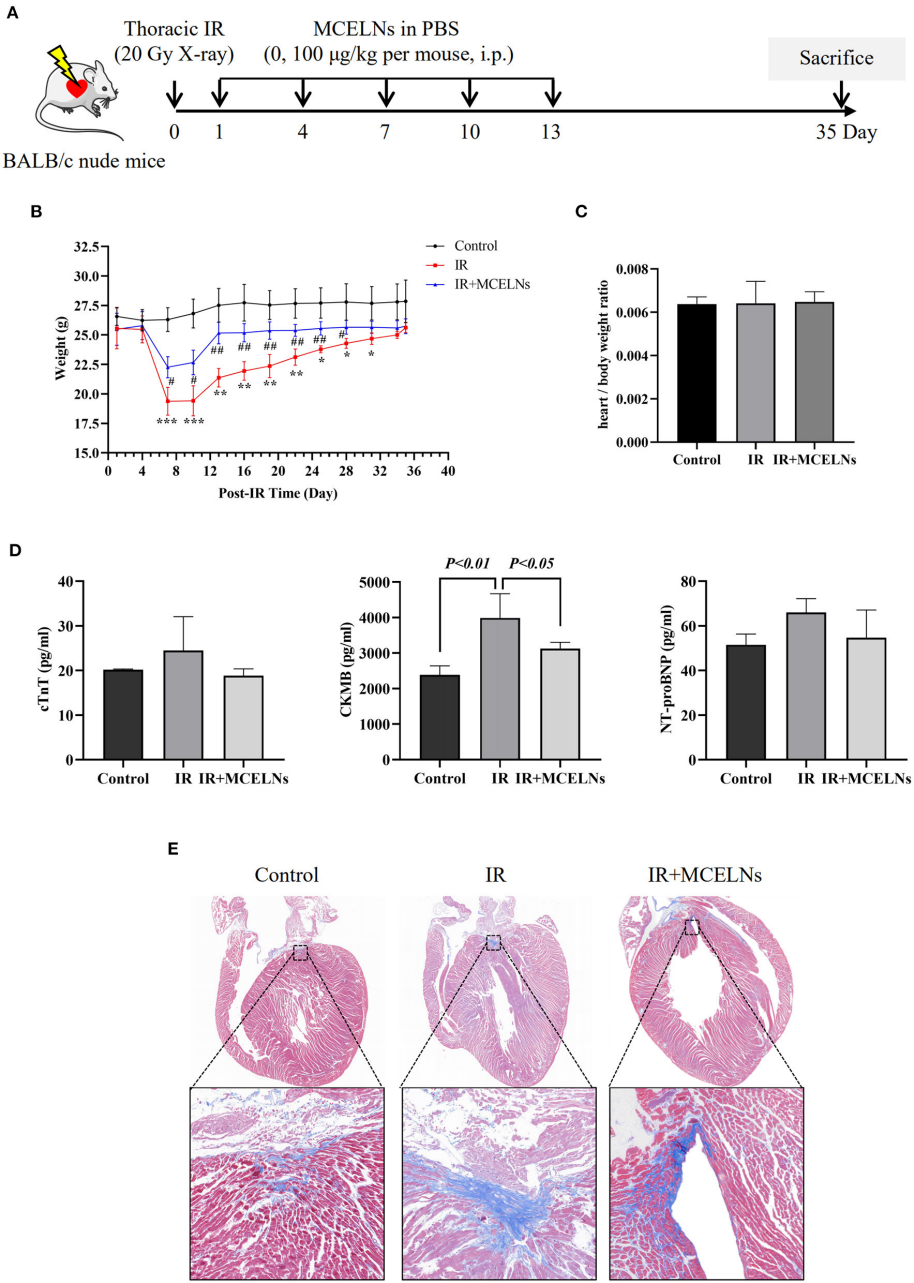
MCELNs exerted *in vivo* protective effect after radiation. **(A)** The generation procedure of thoracic irradiated mice model was illustrated. Briefly, mice were randomly divided into three groups: (1) Control; (2) IR (thoracic irradiation, 20 Gy X-ray, PBS administration post-IR, every other day for 5 times i.p.); (3) IR+MCELNs (thoracic irradiation, 20 Gy X-ray, MCELNs administration post-IR, 100 μg/kg every other day for 5 times, i.p). **(B)** Mice body weight were observed until sacrificed at day 35. **(C)** The ratio of mice heart weight to body weight were calculated. **(D)** Serum samples were collected to detect the concentrations of cardiac injury marker cTnT, CKMB, and NT-proBNP using ELISA kit. Masson trichrome staining **(E)** were performed to analyze the heart fibrosis area in mice. i.p, intraperitoneal injection. The statistical significance was evaluated by one-way ANOVA followed by the Turkey's multiple comparisons test among groups. RM two-way ANOVA followed by the Turkey's multiple comparisons test was used for significance evaluation of repeat measurement of mice body weight. All data were represented as means ± SD (*n* = 4 independent experiments). *P < 0.05, **P < 0.01, ***P < 0.001 vs. Control. ^#^*P* < 0.05, ^##^*P* < 0.01, vs. IR.

To test the radioprotective effect of MCELNs, we generated a thoracic irradiation model (20 Gy X-ray, 100 cGy/min) in BALB/c nude mice by shielding other body areas with lead except for the chest. Mice that received no thoracic radiation exposure were included as a control. Thoracic irradiated mice were then intraperitoneally administrated with MCELNs (100 μg/kg in PBS, IR+MCELNs) or not (PBS, IR) every two days for five times. Mice were sacrificed 35 days after the initial thoracic irradiation (Figure 6A). Compared with the control, thoracic irradiated mice exhibited a significantly declined body weight in the first couple of days (Figure 6B). The MCELNs administration alleviated the reduction in the body weight of thoracic irradiated mice (Figure 6B). However, the heart/body weight ratio was not different among three groups (Figure 6C). For monitoring cardiac injury after radiation, we tested the biomarkers of myocardial injury including cTnT, CKMB, and NT-proBNP. The expression levels of cTnT, CKMB and, NT-proBNP were increased in irradiated mice compared with non-irradiated and MCELNs-treated mice, and only CKMB changes showed statistical significance (Figure 6D). Moreover, the masson trichrome staining of cardiac tissue sections revealed that the extent of fibrosis in irradiated mice were significantly higher than that of control mice. Meanwhile, MCELNs administration diminished the cardiac fibrosis area in thoracic irradiated mice (Figure 6E).

## Discussion

In the present study, we isolated MCELNs from *M. charantia* that applied as traditional folk medicine and identified them as EVs. MCELNs enhanced cell proliferation, reduced cell apoptosis, and mitigated the DNA damage in H9C2 cells exposed to 16 Gy X-ray. These effects might be attributed to the free radical scavenging ability of MCELNs, as evidenced by declining ROS levels and restored mitochondria function in irradiated H9C2 cells. In addition, the ratios of ROS-related proteins that p-AKT/AKT and p-ERK/ERK were recovered in irradiated H9C2 cells. Finally, intraperitoneal injection of MCELNs mitigated myocardial injury and fibrosis in a thoracic mice irradiation model.

Plant-derived extracts, including polysaccharides, flavonoids, phenylpropanoids, stilbenoids, vitamin C, and gallic acid, have emerged as novel radioprotectors due to their potent antioxidant activity (19, 20). In addition, EVs represent a novel strategy for mitigating radiation-induced adverse effects (21). Accarie et al. showed that intravenously injection of human mesenchymal stem cells-derived EVs protected the intestinal epithelium's integrity in a mouse model of acute radiation syndrome (22). Lately, the utility of plant-derived EVs in therapeutic drugs has also gained substantial interest (23, 24). Hence, we intended to isolate EVs from *M. charantia* that its extract polysaccharide had neuroprotective effects against cerebral ischemia/reperfusion injury via scavenging excessive free radicals (9) and enhancing neural stem cells proliferation (10) and differentiation (11). Employing desentity gradient centrifugation, we successfully obtained MCELNs with typical sizes, shapes, and markers (TSG101, CD63, and CD9) identified to EVs. The effectiveness of non-cell therapy depends on its uptake by target cells (25). Correspondingly, *in vitro* confocal imaging displayed that H9C2 cells endocytosed membrane fluorescent dye PKH67 labeled-MCELNs time-dependently.

Cardiomyocytes undergo apoptosis after radiation exposure (18, 26). Exposure to 16 Gy X-ray significantly declined the cell viability and induced apoptosis in H9C2 cells, consistent with Dai et al.'s report (17). Co-culturing irradiated H9C2 cells with MCELNs improved cell proliferation, demonstrated by the elevated ratio of Ki-67 positive H9C2 cells and protein expressions of cell proliferation marker PCNA. Moreover, radiation exposure reduced the expression of Cyclin D1 and Cyclin B1, indicating cell cycle arrest in irradiated H9C2 cells. MCELNs improved Cyclin D1 and Cyclin B1 expression, suggesting recovered cell cycle thus improving H9C2 cells proliferation. In addition, MCELNs suppressed the apoptosis in irradaited H9C2 cells, as evidenced by decreased pro-apopototic proteins (cleavead caspase3 and cleavead PARP) and decreased apoptotic cells. These results suggested that MCELNs prevented radiation-induced toxicity in H9C2 cells *in vitro*.

High doses of radiation cause DNA injury, including base damage, cross-links, single-strand breaks, and double-strand breaks that further alter gene expression, posing genome instability, and cell death (27). Here, we detected the expression of γ-H2A.X, a widely used biomarker of DNA double-strand breaks (28). Radiation exposure significantly upregulated the expression of γ-H2A.X in H9C2 cells, and MCELNs alleviated this damage. Moreover, as the upstream of γ-H2A.X, autophosphorylation of ATM modulates various cellular responses especially initiated DNA double-strand breaks (29). We found that the phosphorylation of ATM significantly increased after radiation exposure. Whereas, MCELNs reversed the abnormal phosphorylated ATM to reduce cardiomyocytes' DNA damage. In all, MCELNs mitigated radiation-induced DNA damage in H9C2 cells.

Mitochondria is a vital organelle in cardiomyocytes that participate in multiple cellular responses. As the primary source of cellular ROS, mitochondria generate appropriate ROS to facilitate cellular immune responses, signal transduction, and apoptosis under normal conditions (30). Radiation disrupts the mitochondria respiratory chain, causing energy metabolism imbalance, generating excessive ROS, initiating cell apoptosis (31). In irradiated H9C2 cells, we observed significantly elevated mitochondria ROS correspondingly. *M. charantia* polysaccharide can clear superoxide, nitric oxide, and peroxynitrite, modulating oxidative stress in neural stem cells (9). Likewise, MCELNs also effectively scavenge radiation-induced excessive mitochondria ROS in H9C2 cells. Standard MMP is the precondition for maintaining mitochondrial oxidative phosphorylation and cellular physiological functions (32). With JC-1 probe staining, we found that MCELNs restored the decreased MMP in irradiated H9C2 cells. Re-balanced mitochondria ROS and MMP may contribute to the protective potentials of MCELNs against RIHD. AKT and ERK signaling pathways have been shown to play vital roles in cell survival and proliferation. And activation of AKT and ERK were associated with cancer radiation resistance. Under stress conditions, excessive ROS generation was reported to reduce the AKT and ERK activation (33). Accordingly, we found significantly reduced p-AKT and p-ERK in irradiated H9C2 cells consistent with Dai et al.'s report (17). MCELNs co-culture recovered the phosphorylation of AKT and ERK. Gu et al. found RhNRG-1β protected the irradiation-induced myocardium injury via activating the ErbB2-ERK-SIRT1 pathway (34). Inhibition of AKT and ERK pathways were also observed in the *in vitro* model of angiotensin II stimulated cardiomyocytes hypetrophy. Ba et al. found allicin activates PI3K/AKT/mTOR and MAPK/ERK/mTOR signaling pathways to inhibit the autophagy process alleviating cardiac hypertrophy (35). Zhang et al. identified FNDC5 mitigates doxorubicin-induced H9C2 cells cardiomyocytes apoptosis via activating AKT (36). Thus, the activation of AKT and ERK signaling pathways might be involved in the protective effects of MCELNs against RIHD. The specific mechanism remains to be further investigated.

Last, we verified the protective effects of MCELNs against RIHD *in vivo*. We generated a mice model of radiation-induced cardiomyopathy by exposing the chest of BALB/c nude mice to 20 Gy X-rays as previously reported (18). In the first couple of days post-IR, the mice's body weight significantly declined, which was mitigated in MCELNs injected mice. However, the body weight of irradiated mice injected with MCELNs or not recovered to the levels comparable to the control mice by the end of observation (35 days post-IR). Due to the unavailability of an electrocardiogram, we failed to assess the cardiac function of mice. Instead, we detected the serum levels of common cardiac injury biomarkers, including cTnT, CKMB, and NT-proBNP. Thoracic RT increased the serum levels of cTnT, CKMB, and NT-proBNP, and MCELNs injection decreased these changes. Moreover, our data showed that CKMB was more sensitive to radiation-induced cardiac injury. Fibrosis is the severe long-term effect of RT that eventually causes cardiac dysfunction and even heart failure (37). We found that MCELNs injection reduced fibrosis in thoracic irradiated mice. Above all, MCELNs may protect cardiac against radiation-induced damage *in vivo*.

There are several limitations in this study. First, cardiomyocytes are the dominant cell type in the heart that we initial investigate the protective effects of MCELNs on them. It would be interesting to investigate whether MCELNs modulate other cardiac cells (endothelial and fibroblast) function in future studies. Second, we just evaluated the uptake of MCELNs by H9C2 cells *in vitro* and did not investigate how the MCELNs were homed to the injured heart. Last but not the least, we did not unravel the role of MCELNs contents (e.g., proteins, microRNAs) in protecting cardiomyocytes from DNA damage and mitochondrial dysfunction.

Collectively, we here successfully isolate EVs-like MCELNs from *M. charantia* and verify their potent cardio-protective effects against radiation *in vitro* and *in vivo*. Plant-derived EVs can access physical barriers, generate less immunogenicity/toxicity, possess inherent cell/tissue targeting capacity, qualifying MCELNs as novel ideal candidates for managing radiation-induced side effects.

## Data Availability Statement

The original contributions presented in the study are included in the article/supplementary material, further inquiries can be directed to the corresponding authors.

## Ethics Statement

The animal study was reviewed and approved by Institutional Animal Care and Use Committee of Xuzhou Medical University.

## Author Contributions

W-WC: conception and design, collection and/or assembly of data, data analysis and interpretation, and manuscript writing. CYe, K-XW, and XY: collection and/or assembly of data, data analysis, and interpretation. P-YZ and KH: collection and/or assembly of data. TL, L-YH, and W-WC: data analysis and interpretation. BG and CYa: financial support. PM: manuscript editing. S-HQ and LL: conception and design, data analysis and interpretation, manuscript writing, financial support, and final approval of manuscript. All authors contributed to the article and approved the submitted version.

## Funding

This work was supported by the National Natural Science Foundation of China (Grant Nos. 81802086, 81860425, and 81802063), Scientific Research Project of Jiangsu Provincial Healthy Commission (Grant No. ZDB2020024), Natural Science Foundation of Jiangsu Province (Grant No. BK20211348), the project of Science and Technology Department of Jiangxi Province (Grant No. 20204BCJ23018), the project of Science and Xuzhou Basic Research Project (Grant No. KC21030), the Specialized Research Fund for Senior Personnel Program (Grant No. D2019028), and the Young Science and Technology Innovation Team (Grant No. TD202005) of Xuzhou Medical University.

## Conflict of Interest

The authors declare that the research was conducted in the absence of any commercial or financial relationships that could be construed as a potential conflict of interest.

## Publisher's Note

All claims expressed in this article are solely those of the authors and do not necessarily represent those of their affiliated organizations, or those of the publisher, the editors and the reviewers. Any product that may be evaluated in this article, or claim that may be made by its manufacturer, is not guaranteed or endorsed by the publisher.
